# Understanding gender differences of people with HIV newly diagnosed or returning to care with advanced HIV disease in Malawi: a qualitative study

**DOI:** 10.1186/s12889-023-17384-y

**Published:** 2023-12-01

**Authors:** Leila Katirayi, Thulani Maphosa, Lloyd Chilikutali, Rachel K Chamanga, Josephine Petersson, Sarah Khatib, Boswell Munthali, Rose Nyirenda, Eddie Matiya, Laywell Nyirenda, Appolinaire Tiam, Lise Denoeud-Ndam

**Affiliations:** 1 Elizabeth Glaser Pediatric AIDS Foundation, Washington, Geneva USA; 2 Elizabeth Glaser Pediatric AIDS Foundation, Lilongwe, Malawi; 3 George Washington University, Washington, DC USA; 4 Department of HIV and AIDS, Ministry of Health, Lilongwe, Malawi; 5 Elizabeth Glaser Pediatric AIDS Foundation, Geneva, Switzerland

**Keywords:** Advanced HIV disease, Antiretroviral therapy, Social norms, Malawi, Treatment fatigue, Counseling, Stigma, Gender

## Abstract

**Background:**

Despite tremendous progress in improving antiretroviral therapy (ART) access, advanced HIV disease (AHD) still remains a challenge globally. Reasons for delayed presentation to care and ART adherence may be affected by gender. We present qualitative study findings on gender differences in decisions for HIV testing and ART initiation/adherence in adults with AHD in Malawi.

**Methods:**

We used a qualitative study design, interviewing 16 men and 16 women aged 18 years and above diagnosed with AHD in sites implementing an optimized package of AHD care, from December 2021-February 2022. We included study participants receiving AHD services for at least two months. We also interviewed 16 lay workers and 16 health care workers supporting people living with AHD. In-depths interviews (IDIs) were conducted in English or Chichewa by trained research assistants using semi-structured interview guides. A short-answer analysis was conducted, and findings were interpreted according to thematic areas.

**Results:**

Both men and women reported stigma as a main barrier influencing their decision to test for HIV and to initiate and adhere to ART. Fear of side effects, insufficient food, and the need for more information were other barriers reported among men and women as well as perceived as barriers by HCWs. Men appear to have tested later for HIV and stated that they were waiting until experiencing significant symptoms before testing. According to clients and HCWs, men were also less inclined to initiate ART after a HIV diagnosis, whereas women were motivated to start treatment to remain healthy and care for the family. Both genders reported that treatment could be delayed if they were feeling healthy. Treatment fatigue was reported among all groups as the main reason to discontinue treatment.

**Conclusions:**

There were similarities and differences between genders in decision-making about HIV care. Concerns about stigma were important reasons for delay in HIV care in both genders. Motivations for accessing HIV treatment and care were different among men and women, pushing the need for gender-tailored counseling services and community messaging that educate both men and women on the benefits of initiating ART early, in turn reducing the number of people presenting with AHD.

**Trial registration:**

NCT05510973, first registration 22/08/2022.

## Background

Malawi has one of the highest HIV prevalence rates in the world, with over 8% of the adult population (aged 15–49 years) living with HIV in 2020 [1, 2]. Among persons aged 15–49 years old, HIV prevalence is 10.3% in females, and 5.7% in males [1]. Despite tremendous progress in improving access to antiretroviral therapy (ART), advanced HIV disease (AHD) still remains a challenge. AHD is defined by the World Health Organization (WHO) as having a CD4 cell count less than 200 cells/mm^3^ and/or stage III or IV disease for children older than 5 years old and adults [3]. All children less than 5 years old on treatment for less than 12 months or children who are unstable on treatment after 12 months are also considered to have AHD [3]. Data from South Africa demonstrates that a third of patients initiated on ART have AHD [4]. Patients classified as ‘late presenters’ typically exhibit symptoms at the time of their HIV diagnosis, often indicating that they delayed testing because they believed HIV treatment was unnecessary unless they felt unwell [5].On the other hand, a growing number of patients re-present to care at an advanced stage of HIV disease following disengagement from treatment [6].

AHD results in increased morbidity and mortality [3]. Patients with AHD are at higher risk of death, even following initiation of ART, with risk increasing with decreasing CD4 cell count [9]. The leading causes of mortality among patients with AHD are tuberculosis (TB), severe bacterial infections, and cryptococcal meningitis [7].

In 2017, WHO published guidelines for the differentiated management of AHD as part of a public health approach with the aim of reducing mortality in patients with AHD [3]. The WHO-recommended package of AHD care includes: (1) prompt initiation of ART in the absence of contraindications; (2) co-trimoxazole prophylaxis; (3) screening for active tuberculosis (TB) and prompt initiation of anti-TB treatment or tuberculosis preventive treatment as indicated, (4) routine screening for Cryptococcus antigen, and curative or pre-emptive therapy as indicated, and (5) differentiated, people-centered approach to ART delivery with intensified adherence support (more intensive follow-up on ART).

Due to their specific needs, patients with AHD experience specific challenges at the facility and community level. There are a shortage of essential AHD diagnostic tests in some health facilities, as well as delay in getting results after tests. Lack of knowledge regarding HIV in the community also serves as a barrier for the utilization of AHD services. Less than 50% of the population in Malawi has comprehensive knowledge about HIV [8]. This makes fidelity to AHD management and care services more challenging. Furthermore, care and treatment for patients presenting with AHD is associated with significantly higher costs to the health system, emphasizing the need for earlier identification and engagement in care for AHD patients [9]. The complexity of optimized treatment of opportunistic infections is a barrier to successful AHD management, and there is need for strong capacity building efforts among HCWs to increase treatment coverage [10].

In 2019, of 154,454 newly initiated patients on ART, 18,183 (11%) presented with AHD in Malawi [11]. AHD has been shown to commonly occur in patients not on ART, accounting for 59% of those with AHD [12]. AHD in Malawi has been described to occur more frequently in males than females, with 12.9% among males compared to 5.6% of female patients and these finding are similar to Kenya and South Africa [12]. To evaluate the reasons for this gender discrepancy, the Elizabeth Glaser Pediatric AIDS Foundation (EGPAF) in Malawi conducted a qualitative study to evaluate potential gender-related factors for delayed access to HIV care and treatment among patients with AHD in Malawi.

## Methods

### Study design

This was a qualitative study design that involved interviewing men and women with AHD and HCWs providing care to AHD patients. The study explored factors that influenced the delay among newly initiated AHD patients decision to test for HIV and initiate treatment, and factors that influenced previously diagnosed patients’ decision to drop out of care. The study explored the role of gender in patients’ decisions.

### Site selection

Four high volume sites supported by EGPAF in the central region in Malawi in Dedza and Ntcheu districts were purposefully selected. Two sites were selected due being a ‘hub’ site; hub sites are larger sites that supports smaller ‘spoke’ sites by providing supplies, technical support and accepting patients with more complicated health problems. Two spoke sites were selected; spoke sites provide more services at the community level and offer more basic care. Sites were also selected to have an equal distribution between urban and rural settings.

### Study population and sample size

The study population was comprised of HIV-positive men and women, HCWs and lay cadres supporting selected health facilities. Men and women were eligible if they were aged 18 years or older, enrolled in AHD services and receiving AHD services for at least two months. HCWs were those involved in HIV care, including laboratory staff, ART nurses and clinicians. HCWs in training were excluded. Lay cadres included Expert Clients (ECs), Adherence Support Officers (ASOs) and other groups involved in AHD care and follow-up. See Table 1 for full list of HCW and lay cadre positions.

**Table 1:** HCWs’ demographic characteristics

Variables		Provider	
**Characteristics**	**Categories**	**HCWs** **N = 16**	**LCs** **N = 16**
Age	Mean age (SD)	34 (8)	39 (8)
Gender	Male	7	6
	Female	9	10
Clinical Position	Nurse Tec	1	0
	Nurse Midwife Tech	5	0
	Reg Nurse	1	0
	Reg N/M	1	0
	Lab Technician	2	0
	Clinician (MA/CO/MD)	6	0
Lay Cadre Position	Expert Client (EC)	0	6
	HIV Diagnostic Assistant (HDA)	0	2
	Adherence Support Officer (ASO)	0	3
	ART Clerk	0	4
	Hospital Attendant	0	1
Years in position	< 1	1	0
	1–5	9	11
	6–10	3	2
	> 10	2	3
	Missing	1	0

The study aimed to interview an equal number of male and female patients, to represent perspectives from both groups. Sixteen in-depth interviews were conducted with male patients, female patients, lay workers and HCWs. Previous research has demonstrated that saturation per homogenous group can be reached at 12 interviews [13].

### Data collection

Interviews with patients collected information about their experiences with AHD services, decisions regarding HIV testing and treatment, facilitators to receive care, challenges experienced, how challenges were addressed and recommendations to improve care. Interviews with HCWs and lay cadres collected information on their experiences providing AHD services, knowledge required for diagnosis and treatment for AHD patients and perceptions about AHD patients.

From December 2021-February 2022, in-depth interviews (IDIs) were conducted with all participant groups and led by RAs who were hired and trained by EGPAF. Training involved orienting RAs to the protocol, standard operating procedures, piloting data collection tools, mastering qualitative data collection methods and human ethics. RAs used semi-structured interview guides. All IDIs were audio-recorded and completed in English or Chichewa, depending on participant preference. IDIs were transcribed as soon as possible after data collection to minimize recall errors and improve accuracy. Descriptions of participant’s non-verbal responses were also documented in transcripts.

Male and female study participants were recruited using convenience sampling after attending clinical visits. HCWs identified eligible patients and once written consent was obtained, IDIs were conducted on site by RAs. HCWs were recruited by speaking to the health facility’s nurse in-charge to identify eligible HCWs. To recruit lay counselors the health facility’s nurse-in-charge was informed of the eligibility criteria and asked to identify those that were the most involved in supporting AHD patients. Greater emphasis was put on recruiting ECs as they are generally more involved in counseling patients and visiting AHD patient’s homes.

### Analysis

The recorded IDIs were translated from Chichewa to English. A short-answer analysis was conducted, consisting of compiling responses for each question into a Word document. Compiled responses were analyzed for overarching themes and findings were summarized in a separate summary document. Textual data was carefully read to identify recurrent patterns and themes. Data were analyzed to explore differences between male and female patients, and understand their different journeys through the continuum of care. Text excerpts were identified that are representational of themes identified. The research team interpreted the findings according to thematic area and compiled results to be shared with the study team. The results of the qualitative analysis were shared with the EGPAF Quality Improvement (QI) team to help strengthen implementation of the AHD package of services.

## Results

### Patients’ and HCWs’ demographic characteristics

The mean age for male patients was 45 years and 39 years for female patients. While most male patients were married, most female patients were divorced, widowed or separated. The majority of men and women had primary education. The majority of female patients had been recently diagnosed with HIV status and started treatment. See Table 2.

**Table 2:** Male and female patients’ demographic characteristics

Variables		Gender	
**Characteristics**	**Categories**	**Male** **N = 16**	**Female** **N = 16**
Age	Mean age (SD)	45 (9)	39 (8)
	Median age (IQR)	40 (37–53)	40 (35–46)
Marital status	Married with Partner	9	6
	Single	1	0
	Divorced/Separated/Widow	4	10
	*Missing*	*2*	0
Education	Primary	10	14
	Secondary and above	4	2
	*Missing*	*2*	0
Discovered HIV Status in Last few Visits	Yes	6	10
	No	8	6
	*Missing*	2	0
Years Since Tested Positive (years)	0–1 Year	7	12
	2–5 Years	3	0
	5 + Years	4	4
	*Missing*	*2*	2
Years on ART	0–1 Year	7	11
	2–5 Years	3	2
	5 + Years	4	3
	*Unknown*	*2*	0

The majority of health care providers were female and had between 1 and 5 years of experience in their current position. See Table 1.

The study results have been organized by the different stages of HIV care, including HIV testing (for the newly diagnosed), treatment initiation, and retention. The last section provides recommendations to improve AHD care.

### Delayed HIV testing among the newly diagnosed

HCWs reported patients often delayed HIV testing due to fear of stigma and discrimination from the patients’ communities, and disagreements with their families or partners regarding HIV testing.

*They are afraid that news of their infection will spread in the villages, and also, it’s because some of them do not know the importance of early testing and treatment so it is very vital that they reach out to people*. 1816 - HW − 001 - Lizulu (Female HCW, Nurse Midwife Tech, age 27).

HCWs and LCs said many newly diagnosed patients did not suspect they had HIV infection and did not feel the need to come to the clinic. Some of these patients believed their illness were secondary to more common problems, such as malaria.

*Most of the times they tell us that they did not expect to be diagnosed with such diseases, they think it’s merely Malaria or that they have been overworking so in the midst of buying painkillers and wondering what to do at that time they notice that the pains are gone, and after a short time it comes back again and in so doing time is running out.* 1723 - LC − 001 - Mtendere (Female HCW, ART Clerk, age 48).

Some patients attempted to treat symptoms resulting in delayed HIV testing. Some patients did not consider HIV to be a cause for their symptoms.

*When I got sick it happened that I would buy different kinds of medication to drink and it also happened that I had cough and I was coughing persistently and I had no strength I was so weak that time I was just weak, I would buy medications and take but wouldn’t get well.* 1816-FP-001- Lizulu. (Female patient, age 36)

Men were less likely to come for an HIV test. Women were more likely than men to seek healthcare and treatment, as women are more likely to be visiting clinics for prenatal care and care for children.

*Men come here for testing and treatment when they are already at an advanced stage of HIV but women try their best to come for HIV testing and treatment early. You will also notice that even after HIV testing, they are men who do not disclose their HIV status to their wives which if different from women, who disclose their HIV statuses to their husbands.* 1836 - HW − 001 – St Theresa (Male HCW, Lab Technician, age 50).

### Treatment initiation

Because some newly diagnosed patients did not feel very sick, they did not feel any urgency to initiate treatment after learning their HIV status.

*My body seemed to be in good health. I was strong in a nutshell, I just felt like my body was in good shape, I wasn’t sick.* 1723-FP-002-Mtendre (Female patient, age 26).

HCWs and LCs said that newly diagnosed patients presenting with AHD discussed logistical challenges, including, transportation (costs, methods & distance), living or working out of the country, concerns of having to pay for services, or being too busy.

*Most of the times there are some AHD patients that travel long distances to seek the services that are mostly found here at the district hospital. Some even travel a distance of over 30 km….Of course, some would want to seek the treatment as early as possible but the distance hinders them.* 1609 - HW − 001 - Mchinji (Male HCW, Clinician, age 31).

Initiating treatment meant accepting one’s HIV status, and sometimes resulted in delayed treatment initiation, especially among men. Men struggled more with disclosure and the shame associated with HIV, which may discourage them from testing.

*I made the choice of coming to [treatment facility name] for my ARV refill and treatment because of how patients are insulted at our village hospital. The insult that the patients get is about disclosing your HIV status to other people.* 1816-MP-001-Lizulu (Male patient, age 40).

Men were more concerned about being ‘seen’ at the facility and others learning their status, resulting in them seeking healthcare outside their community. Men were also more likely to delay treatment due to working outside of the country.

*Let’s start with the men, when men are asked, they say they were in Lilongwe to work and run out of medicine while there and do not have transport to come back. I have met about three men who have given that reason. Whereas with women mostly they give reasons related to marriage. Such as they were called to Mzuzu by their husbands and they went there with three months-worth of medicine but they ended up staying there for six months because they were not being given transport. Most people give reasons as such.* 1836 - HW − 002 – St Theresa (Male HCW, Nurse Midwife Tech, age 27).

HCWs reported that women are more accepting of their HIV status and ready to start treatment if pregnant to protect their unborn child, or if not pregnant, to ensure that she is alive to take care of her children.

*For instance, a woman is HIV positive and she’s pregnant is she supposed to skip? She doesn’t skip because she wants to care for the baby so she is just courageous and tells herself that this is it, I will take the medication consistently because of my unborn child.* 1609 - LC − 001 - Mchinji (Female LC, Adherence Support Officer, age 31).

### Treatment retention

Overall, HCWs and LCs said that women are more likely than men to adhere to ART,which HCWs believed is because women are more interested in protecting their health, especially when pregnant and wanting to protect their unborn child.

Challenges that resulted in both male and female patients delaying or stopping treatment included becoming tired of taking their medications, side effects of the medications, or after hearing rumors of possible side effects. Patients who experience side effects were more likely to stop treatment.

*Most of them come and complain that the ART drugs are making them vomit daily. Some also complain that the ART drugs make them easily forget things. These are the reasons that make most of HIV patients not to return to the facility to get their drugs when they run out.* 1609 - LC − 004 - Mchinji (Female LC, HIV Diagnostic Technician, age 27).

Many patients said that lack of food caused them to stop taking medication. They explained that food was necessary because otherwise medication would make them feel unwell, causing them to be less likely to adhere. This sentiment was discussed equally among men and women.

.*If I don’t have any food to eat, I skip taking the medication because drugs on an empty stomach might be problematic for me*.1836-MP-002-St Theresa (Male patient, age 38).

Another challenge discussed by HCWs was patients stopping treatment when they started to feel better.

*Most people adhered to drugs when they first got diagnosed with HIV, but when they notice that they have gained strength and they are getting better, they feel that the drugs have made them feel better. And the first thing that they do is skip taking their drugs.* 1836-LC-004-St Theresa (Female LC, Expert Client, Age 46).

HCWs and female patients reported male partners preventing their female partners from accessing ART.

*I stopped because of my marriage then, that’s the reason we divorced he would deny me from going to the hospital, he said I wanted people to mock him about the disease because they would see me going to the ART so he tore my card and disposed it in the toilet together with the medication and he also beat me up so I took some time without taking the medication.* 1836-FP-003- St Theresa (Female patient, age 40).

HCWs reported that many were confused to learn that they had AHD, as they reported that they had been adhering to medication. HCWs reported that women seemed more surprised them men to learn that they had AHD. Men were more accepting of their AHD status.

*HCWs said that previously diagnosed HIV-positive patients are stressed, sad, and disappointed to hear that they have developed AHD, and many are confused as to why the treatment is not working for them, especially when they claim they have been adhering to ART. HCWs/LCs added that counseling helps these patients to accept.* 1723 - HW − 003 - Mtendere (Female HCW, Clinician, age 29).

Patients reported that sometimes they occasionally missed their medication, but that they did not go long periods without treatment. Other patients were surprised to learn they had AHD, as they were not experiencing increased illness.

*Because I was not feeling any body pains or anything, so when I was told that I had advanced HIV, because of the state of your immunity you are very sick. I was surprised to hear that I was sick, because I was feeling ok.* 1836-FP-003- St Theresa (Female patient, age 40).

### Recommendations to improve AHD services

Many patients described needing additional information from the HCWs on what advanced HIV is, treatment side effects, clinic appointment schedules, and living with AHD.

*I would have liked to receive such information, I would not have resisted because I could have known that the drugs are bringing in such results so that if anything, health care workers should be aware of how they could help me.*1836-MP-003-St Theresa (Male patient, age 60).

Many patients also requested more counseling from HCWs on medication adherence since they did not feel well informed about medication changes with AHD and alternative medication options.

*I would have liked to get more information on the importance of continuing taking drugs because if we stop taking the drugs our advanced HIV status will worsen which will lead to death. As such, we are supposed to follow the procedures of taking drugs because we are losing our friends who are dying because they stopped taking drugs.* 1816-FP-004-Lizulu (Female patient, age 46).

HCWs said patients often need additional counseling on misinformation heard throughout their community, as well as viral load interpretations. This was more common among women.

*Other topics that AHD patients have questions on are the facts that when they start taking ARVs, some believe that they are not going to conceive and give birth. That is what they tell each other. Others tell their fellow HIV patients that even if they try to conceive, they will give birth to a disabled child so some HIV patients do not understand this. The other topic is about discordant couples that they have discovered that only the husband is HIV positive while the wife is HIV negative, on that, it is a challenging topic again.* 1837 - HW − 004 - St Theresa (Male HCW, Reg, age 47).

HCWs said AHD patients need additional information on how to properly take their medication as patients believe they can take their medication when they want and can stop when they are feeling well again.

*There is a need to add more information. Even though we try to tell them about it, patients feel that we are lying to them. When they return to their homes and start feeling better, they feel that they are cured. That is the first message that we should emphasize.* 1836 - HCW − 004 - St Theresa (Male HCW, Reg, age 47).

Patients requested additional home visits. LCs added that additional resources are needed to support counseling and home visits.

*If possible sometimes visiting us in our home is good, if it’s not possible to visit, we can communicate over the phone which is pretty much the same with visiting.* 1609-MP-003-Mchinji (Male patient, age 37).

To improve experience during facility visits, many patients wanted shorter waiting times and larger waiting spaces. They also expressed need for a more private location to pick up medications in the facility. The need for more privacy and discretion was mentioned more frequently among the men.

*I think the place can be improved by building a shelter so that we can be waiting for our services at a private place.* 1836-MP-004-St Theresa (Male patient, age 39).

Male and female patients wanted to receive larger supplies of medication to reduce trips to the facility.

## Discussion

### HIV testing

Gender influenced patients’ HIV experiences starting with HIV testing through remaining in care. Patients and HCWs both discussed that men struggled more with HIV testing. Many men delayed HIV testing, citing concerns about the impact on their livelihood. Men are the heads of families and have higher social status than women in the community, and therefore they may be afraid that being seen at the facility many negatively impact their status [14]. Some studies found that men questioned their ability to be a ‘head of the household’ if HIV-positive [15].

Women were more accepting of testing for HIV, and often received testing through antenatal care. Women expressed more concerns about disclosure of their HIV status, rather than HIV testing itself. Women’s main concern regarding disclosure is possible intimate partner violence and fear of abandonment [16, 17]. Previous research has documented that women may often test for HIV secretly and if found positive, will return with male partners for HIV testing, pretending to not know their HIV status. This strategy helps to avoid blame or violence [18].

Both genders discussed not suspecting an HIV infection, because they did not feel sick enough to suspect an HIV infection. Previous literature has discussed at length that patients may not test for HIV, because they did not think HIV was a possibility, or believed their symptoms were related to a common illness such as malaria. This was true among our study’s participants, who explained that they had delayed testing for HIV because they believed the symptoms were related to common illnesses.

Stigma was commonly cited as a main concern regarding HIV testing among both genders, with concerns about how a HIV status would affect one’s social status and role in society. Most of the research regarding late HIV testing and ART initiation have discussed stigma being a significant concern [17]. In a study in Ethiopia, participants who reported fear of stigma were 4.4 times more likely to present late compared to those who did not [19].

### ART initiation

HCWs in this study and previous literature reported that female patients are more likely to initiate ART after an HIV diagnosis than men. Women are more likely to initiate ART rapidly to ensure their own health and be able to care for their family’s health [17]. However, women’s ability to access care is often impacted by their male partners. Previous research has discussed that traditional gender norms can significantly limit women’s capacity to engage in care, since many women need male partner permission to access the resources to attend clinics [20].

Men are less likely to initiate ART immediately after diagnosis due to concerns about loss of employment if an HIV status becomes known, and concerns about requests for time off to attend the facility being denied [16, 17, 21]. In Malawi older men fear loss of respect from younger men, relatives and wives [22].

Men who wish to access ART without potential disclosure of one’s HIV status often register at the health facilities far from their community to maintain privacy [23]. Other literature has cited concerns about men not trusting HCWs to maintain secrecy regarding one’s HIV status [16], but this was not reported in our study.

Feeling ‘healthy’ affected both men and women’s initiation of ART. Delaying treatment if one felt ‘healthy’ was reported in this study and previous research [23, 24]. One study reported that nearly all late presenters waited until they were ill and symptomatic to initiate treatment [17]. A study in Zimbabwe and Uganda found that ART is not considered ‘necessary’ if patients could successfully manage their symptoms [6.

This study and previous research have documented how patients attempt to treat poor health through alternative therapies, including herbal treatments and products from the pharmacist. A study in Zambia reported that while some men were unwilling to test for HIV, they were comfortable attempting to ‘self-care’ for their symptoms with herbal and conventional non-HIV medication to deal with symptoms of opportunities infections, such as periodic episodes of diarrhea, rash, sore throats and coughing [25]. A systematic review exploring the determinants of adherence to ART among HIV-positive adults in sub-Saharan Africa also reported the use of traditional/herbal medicine to be a significant determinant for non-adherence to ART [26].

### Retention

Challenges with logistics were experienced in our study by both genders, but the challenges differed. Men experienced logistical challenges related to employment, including concerns about accessing the facility during working hours and working outside the country [22]. Female participants reported more challenges with costs and logistics. Women are more concerned about care of their children and schools fees [18].

Challenges with side effects were reported equally across the genders. Previous research has discussed that many individuals are aware of side effects associated with older medications [17]. Our study participants also reported tiring of daily medication. Lack of food was also reported as a challenge by both genders.

HCWs reported that both genders stopped treatment when feeling better. Treatment fatigue has been documented in other studies [18]. To address this challenge, additional counseling is needed. Despite some participants reporting treatment fatigue, study participants that had been previously diagnosed, and were on treatment, were surprised to learn that they had AHD, as they felt they adhered well to treatment, rarely missing their medications.

### Recommendations

AHD patients require additional support. Both patients and HCWs recommended increased home visits, to help care for the patients too ill to travel to the health facility. Patients expressed a need for additional information about the medication, potential side effects, clinic appointment schedules and living with AHD. HCWs felt that patients needed more information about how to take the medication, to continue with the medication when feeling better and to address misinformation shared in the community.

Counseling needs to help patients overcome feelings of fear and assist them in their decision to initiate ART [23]. There is a need to address men’s poor health care seeking behavior, specifically regarding HIV testing and treatment [22]. HIV messaging needs to target both genders with messages that resonate with them and their societal roles and norms. Hegemonic notions of a ‘real man’ need to be addressed when addressing gender in HIV care and treatment programs [15]. A study in Malawi encouraged community interventions which encourage older men with community seniority to endorse early HIV testing and treatment services and improve men’s networks [2].

Previous research has documented that men view the health facility as a female focused space [27]. To ensure that men feel comfortable accessing HIV testing and treatment services, in 2017, EGPAF Malawi introduced male friendly clinics, these clinics provided discreet HIV testing services (among other health services) to men at health care facilities on Saturday mornings, so that men could access the facility outside work hours and during non-peak hours. Services were also provided by male HCWs. The male friendly clinics reported high acceptance rate for HIV testing (94%). Because the clinics offered a variety of other health services, they also reduced the stigma regarding utilizing HIV services (all services were provided in one space) [28].

Another study conducted in Cameroon found that men’s perceptions about HIV testing and treatment vary by generation. The study found that older men are more fearful of HIV testing, while younger men were more accepting. The study also found that the generations preferred to receive information through different mechanisms, with younger men being open to receiving information from social media and various sources, while older men preferred to just receive information from HCWs [29]. Improving men’s knowledge of HIV testing and treatment will not only improve health outcomes for men, but will likely improve health outcomes for their female partners as well, as previous literature has documented that spousal support increases ART adherence [30].

Campaigns promoting HIV testing have also shown success with reducing late presenters. One study in Mozambique found that patients diagnosed in 2020 and 2019 had an 85% and 73% lower chance of presenting late to ART when compared with those diagnosed in 2015 [31].

### Next steps

In the context of Malawi, our study’s findings shed light on the socio-economic and cultural factors that influence HIV care decisions among adults with AHD. Notably, the economic challenges Malawians face, including food insecurity and concerns about employment, emerge as critical barriers to early HIV testing and treatment initiation, particularly among men. Furthermore, deeply entrenched cultural gender norms play a substantial role, with men’s roles as providers and women’s roles as caregivers impacting their attitudes toward care engagement. These findings underscore the need for tailored interventions and targeted messaging that consider the societal roles and norms associated with gender.

To improve HIV care and treatment outcomes, efforts should focus on addressing men’s poor healthcare-seeking behavior, promoting early testing and treatment, enhancing counselling services, and providing comprehensive information about medication, side effects, and living with HIV [14, 32–34]. Addressing these challenges requires community-based interventions, endorsement by respected leaders, tailored education and awareness campaigns, and a nuanced understanding of intersectionality among socio-economic and cultural factors. These discussions underscore the need for holistic, culturally sensitive approaches to HIV care in Malawi, with a focus on reducing disparities and increasing accessibility [31, 35–37].It is important to acknowledge a few limitations in this study. We only collected data among individuals already receiving AHD services, those not receiving care may experience other challenges which have not been documented in this study. Generalizability may also be limited, given the specific study population, and there’s a risk of recall and social desirability bias affecting the accuracy of participants’ responses. Nevertheless, this study exhibits several strengths, notably its comprehensive examination of gender-related issues in HIV care through the inclusion of both men and women with AHD as well as healthcare workers. This diverse representation enhances the breadth and applicability of the findings, making them relevant to public health policy and program development in Malawi and similar settings.

## Conclusion

In summary, this study enriches the literature by offering a nuanced examination of gender-specific factors affecting HIV care decisions in the context of AHD in Malawi. Its findings have practical implications for public health interventions and highlight the importance of addressing both common and gender-specific barriers to improve HIV testing, treatment initiation, and adherence, ultimately leading to better health outcomes for individuals living with HIV.

## Data Availability

Data supporting the conclusions of this article are available upon reasonable request to the corresponding author.

## List of abbreviations

AHD: Advanced HIV disease
ART: Antiretroviral treatment
EGPAF: Elizabeth Glaser Pediatric AIDS Foundation
EC: Expert client
HCW: Health care worker
HDA: HIV diagnostic assistant
IDI: In-depth interview
IRB: Institutional review board
LC: Lay counselor
RA: Research assistant
TB: Tuberculosis
WHO: World Health Organization
